# Supplementary material for: Nosocomial Vs. Community-Acquired Infective Endocarditis in Spain: Location, Trends, Clinical Presentation, Etiology, and Survival in the 21st Century
Source: J Clin Med. 2019 Oct 22;8(10):1755. doi: 10.3390/jcm8101755 (PMC6833111; doi:10.3390/jcm8101755)
Supplement: Supplementary file 1 [file jcm-08-01755-s001.pdf]

Supplementary Table 1. ***ICD-9*** **Codes for Comorbidities and Causative Organisms**

Baseline Comorbidities	ICD-9 CM Codes
**Hypertension**	Diagnosis Codes<br>401, 401.0, 401.1, 401.9, 402, 402.0, 402.00, 402.01, 402.1, 402.10, 402.11, 402.9, 402.90, 402.91, 403, 403.0, 403.00, 403.01, 403.1, 403.10, 403.11, 403.9, 403.90, 403.91, 404, 404.0, 404.00, 404.01, 404.02, 404.03, 404.1, 404.10, 404.11, 404.12, 404.13, 404.9, 404.90, 404.91, 404.92, 404.93, 405, 405.0, 405.01, 405.09, 405.1, 405.11, 405.19, 405.9, 405.91, and 405.99
**Complicated diabetes**	Diagnosis Codes<br>250.4, 250.40, 250.41, 250.42, 250.43, 250.5, 250.50, 250.51, 250.52, 250.53, 250.6, 250.60, 250.61, 250.62, 250.63, 250.7, 250.70, 250.71, 250.72, 250.73, 250.9, 250.90, 250.91, 250.92 and 250.93
**Coronary Artery Disease**	Diagnosis Codes<br>411, 411.0, 411.1, 411.8, 411.81, 411.89, 412, 413, 413.0, 413.1, 413.9, 414, 414.0, 414.00, 414.01, 414.02, 414.03, 414.04, 414.05, 414.06, 414.07, 414.2, 414.3, 414.4, 429.5, 429.6, 429.7, 429.71, and 429.79
**Peripheral vascular disease**	440.0, 440.1, 440.2, 440.20, 440.21, 440.22, 440.23, 440.24, 440.29, 440.3, 440.30, 440.31, 440.32, 440.4, 440.8, 440.9, 447.1, 996.1, 996.62, 996.74
**Chronic Obstructive pulmonary disease**	Diagnosis codes (index or prior admissions)<br>491, 491.0, 491.1, 491.2, 491.20, 491.21, 491.22, 491.8, 491.9, 492, 492.0, 492.8, 493, 493.0, 493.00, 493.01, 493.02, 493.1, 493.10, 493.11, 493.12, 493.2, 493.20, 493.21, 493.22, 493.8, 493.80, 493.81, 493.82, 493.9, 493.90, 493.91, 493.92, 494, 494.0, 494.1 and 496
**Renal Disease**	Diagnosis codes (index or prior admissions)<br>403, 403.0, 403.00, 403.01, 403.1, 403.10, 403.11, 403.9, 403.90, 403.91, 404, 404.0, 404.00, 404.01, 404.02, 404.03, 404.1, 4041.0, 404.11, 404.12, 404.13, 404.9, 404.90, 404.91, 404.92, 404.93, 585, 585.1, 585.2, 585.3, 585.4, 585.5, 585.9, 586
**Liver Disease**	Diagnosis codes (index or prior admissions)<br>070, 070.0, 070.1, 070.2, 070.20, 070.21, 070.22, 070.23, 070.3, 070.30, 070.31, 070.32, 070.33, 070.4, 070.41, 070.42, 070.43, 070.44, 070.49, 070.5, 070.51, 070.52, 070.53, 070.54, 070.59, 070.6, 070.7, 070.70, 070.71, 070.9, 456, 456.0, 456.1, 456.2, 456.20, 456.21, 456.3, 456.4, 456.5, 456.6, 456.8, 570, 571, 571.0, 571.1, 571.2, 571.3, 571.4, 571.40, 571.41, 571.42, 571.49, 571.5, 571.6, 571.8, 571.9, 572, 572.0, 572.1, 572.2, 572.3, 572.4, 572.8, 573, 573.0, 573.1, 573.2, 573.3, 573.4, 573.5, 573.8, 573.8, 789.1, 789.5, 789.51, 789.59

**Peptic ulcer**	Diagnosis Codes<br>530.20, 530.21, 530.85, 531, 531.0, 531.00, 531.01, 531.10, 531.11, 531.3, 531.30, 531.31, 531.4, 531.40, 531.41, 531.5, 531.50, 531.51, 531.9, 531.90, 531.91, 532.0, 532.00, 532.01, 532.1, 532.10, 532.11, 532.2, 532.20, 532.21, 532.3, 532.30, 532.31, 532.4, 532.40, 532.41, 532.5, 532.50, 532.51, 532.6, 532.60, 532.61, 532.7, 532.70, 32.71, 532.9, 532.90, 532.91, 533, 533.0, 533.00, 533.01, 533.1, 533.10, 533.11, 533.2, 533.20, 533.21, 533.3, 533.30, 533.31, 533.4, 533.40, 533.41, 533.5, 533.50, 533.51, 533.6, 533.60, 533.61, 533.7, 533.70, 533.71, 533.9, 533.90, 533.91, 534, 534.0, 534.00, 534.01, 534.1, 534.10, 534.11, 534.2, 534.20, 534.21, 534.3, 534.30, 534.31, 534.4, 534.40, 534.41, 534.5, 534.50, 534.51, 534.6, 534.60, 534.61, 534.7, 534.70, 534.71, 534.9, 534.90, 534.91
**Cancer**	Oropharyngeal cancers<br>Diagnosis codes (index or prior admissions)<br>140, 140.0, 140.1, 140.3, 140.4, 140.5, 140.6, 140.8, 140.9, 141, 141.0, 141.1, 141.2, 141.3, 141.4, 141.5, 141.6, 141.8, 141.9, 142, 142.0, 142.1, 142.2, 142.8, 142.9, 143,143.0, 143.1, 143.8, 143.9, 144, 144.0, 144.1, 144.8, 144.9, 145, 145.0, 145.1, 145.2,145.3, 145.4, 145.5, 145.6, 145.8, 145.9, 146, 146.0, 146.1, 146.2, 146.3, 146.4, 146.5,146.6, 146.7, 146.8, 146.9, 147, 147.0, 147.1, 147.2, 147.3, 147.8, 147.9, 148, 148.0, 148.1, 148.2, 148.3, 148.8, 148.9, 149, 149.0, 149.1, 149.8 and 149.9<br><br>Gastrointestinal cancers<br>Diagnosis codes (index or prior admissions)<br>150, 150.0, 150.1, 150.2, 150.3, 150.4, 150.5, 150.6, 150.8, 150.9, 151.0, 151, 151.1, 151.2, 151.3, 151.4, 151.5, 151.6, 151.8, 151.9, 152, 152.0, 152.1, 152.2, 152.3, 152.8, 152.9, 153, 153.0, 153.1, 153.2, 153.3, 153.4, 153.5, 153.6, 153.7, 153.8, 153.9, 154, 154.0, 154.1, 154.2, 154.3, 154.8, 155, 155.0, 155.1, 155.2, 156, 156.0, 156.1, 156.2, 156.8, 156.9, 157, 157.0, 157.1, 157.2, 157.3, 157.4, 157.8, 157.9, 158, 158.0, 158.8, 158.9, 159, 159.0, 159.1, 159.8 and 159.9<br><br>Respiratory tract cancers<br>Diagnosis codes (index or prior admissions)<br>160, 160.0, 160.1, 160.2, 160.3, 160.4, 160.5, 160.8, 160.9, 161, 161.0, 161.1, 161.2, 161.3, 161.8, 161.9, 162, 162.0, 162.2, 162.3, 162.4, 162.5, 162.8, 162.9, 163, 163.0, 163.1, 163.8, 163.9, 164, 164.0, 146.1, 146.2, 146.3, 146.8, 164.9, 165, 165.0, 165.8, and 165.9<br><br>Bone and connective tissue cancer<br>Diagnosis codes (index or prior admissions)<br>170, 170.0, 170.1, 170.2, 170.3, 170.4, 170.5, 170.6, 170.7, 170.8, 170.9, 171, 171.0, 171.2, 171.3, 171.4, 171.5, 171.6, 171.7, 171.8, 171.9, 172, 172.0, 172.1, 172.2, 172.3, 172.4, 172.5, 172.6, 172.7, 172.8, 172.9, 173, 173.0, 173.00, 173.01, 173.02, 173.09, 173.1, 173.10, 173.11, 173.12, 173.19, 173.2, 173.20, 173.21, 173.22, 173.29, 173.3, 173.30, 173.31, 173.32, 173.39, 173.4, 173.40, 173.41, 173.42, 173.49, 173.5, 173.50,173.51, 173.52, 173.59, 173.6, 173.60, 173.61, 173.62, 173.69, 173.7, 173.70, 173.71, 173.72, 173.79, 173.8, 173.80, 173.81, 173.82, 173.89, 173.9, 173.90, 173.91, 173.92, 173.99, 174, 174.0, 174.1, 174.2,

	174.3, 174.4, 174.5, 174.6, 174.8, 174.9, 175, 175.0, 175.9, 176, 176.0, 176.1, 176.2, 176.3, 176.4, 176.5, 176.8 and 176.9<br><br>Genitourinary cancers<br>Diagnosis codes (index or prior admissions)<br>179, 180, 180.0, 180.1, 180.8, 180.9, 181, 182, 182.0, 182.1, 182.8, 183, 183.0, 183.2, 183.3, 183.4, 183.5, 183.8, 183.9, 184, 184.0, 184.1, 184.2, 184.3, 184.4, 184.8, 184.9, 185, 186, 186.0, 186.9, 187, 187.1, 187.2, 187.3, 187.4, 187.5, 187.6, 187.7, 187.8, 187.9,188, 188.0, 188.1, 188.2, 188.3, 188.4, 188.5, 188.6, 188.7, 188.8, 188.9, 189, 189.0,189.1, 189.2, 189.3, 189.4, 189.8 and 189.9<br><br>Lymphoid cancers<br>Diagnosis codes (index or prior admissions)<br>200, 200.0, 200.00, 200.01, 200.02, 200.03, 200.04, 200.05, 200.06, 200.07, 200.08, 200.1, 200.10, 200.11, 200.12, 200.13, 200.14, 200.15, 200.16, 200.17, 200.18, 200.2, 200.20, 200.21, 200.22, 200.23, 200.24, 200.25, 200.26, 200.27, 200.28, 200.3, 200.30, 200.31, 200.32, 200.33, 200.34, 200.35, 200.36, 200.37, 200.38, 200.4, 200.40, 200.41, 200.42, 200.43, 200.44, 200.45, 200.46, 200.47, 200.48, 200.5, 200.50, 200.51, 200.52, 200.53, 200.54, 200.55, 200.56, 200.57, 200.58, 200.6, 200.60, 200.61, 200.62, 200.63, 200.64, 200.65, 200.66, 200.67, 200.68, 200.7, 200.70, 200.71, 200.72, 200.73, 200.74, 200.75, 200.76, 200.76, 200.77, 200.78, 200.8, 200.80, 200.81, 200.82, 200.83, 200.84, 200.85, 200.86, 200.87, 200.88, 201, 201.0, 201.00, 201.01, 201.02, 201.03, 201.04, 201.05, 201.06, 201.07, 201.08, 201.1, 201.10, 201.11, 201.12, 201.13, 201.14, 201.15, 201.16, 201.17, 201.18, 201.2, 201.20, 201.21, 201.22, 201.23, 201.24, 201.25, 201.26, 201.27, 201.28, 201.4, 201.40, 201.41, 201.42, 201.43, 201.44, 201.45, 201.46, 201.47, 201.48, 201.5, 201.50, 201.51, 201.52, 201.53, 201.54, 201.55, 201.56, 201.57, 201.58, 201.6, 201.60, 201.61, 201.62, 201.63, 201.64, 201.65, 201.66, 201.67, 201.68, 201.7, 201.70, 201.71, 201.72, 201.73, 201.74, 201.75, 201.76, 201.77, 201.78, 201.9, 201.90, 201.91, 201.92, 201.93, 201.94, 201.95, 201.96, 201.97, 201.98, 202, 202.0, 202.00, 202.01, 202.02, 202.03, 202.04, 202.05, 202.06, 202.07, 202.08, 202.1, 202.10, 202.11, 202.12, 202.13, 202.14, 202.15, 202.16, 202.17, 202.18, 202.2, 202.20, 202.21, 202.22, 202.23, 202.24, 202.25, 202.26, 202.27, 202.28, 202.3, 202.30, 202.31, 202.32, 202.33, 202.34, 202.35, 202.36, 202.37, 202.38, 202.4, 202.40, 202.41, 202.42, 202.43, 202.44, 202.45, 202.46, 202.47, 202.48, 202.5, 202.50, 202.51, 202.52, 202.53, 202.54, 202..55, 202.56, 202.57, 202.58, 202.6, 202.60, 202.61, 202.62, 202.63, 202.64, 202.65, 202.66, 202.67, 202.68, 202.7, 202.70, 202.71, 202.72, 202.73, 202.74, 202.75, 202.76, 202.77, 202.78, 202.8, 202.80, 202.81, 202.82, 202.83, 202.84, 202.85, 202.86, 202.87, 202.88, 202.9, 202.90, 202.91, 202.92, 202.93, 202.94, 202.95, 202.96, 202.97, 202.98, 203, 203.0, 203.00, 203.01, 203.02, 203.1, 203.10, 203.11, 203.12, 203.8, 203.80, 203.81 and 203.82<br><br>Hematologic cancers<br>Diagnosis codes (index or prior admissions)<br>204, 204.0, 204.00, 204.01, 204.02, 204.1, 204.10, 204.11, 204.12, 204.2, 204.20, 204.21, 204.22, 204.8, 204.80, 204.81, 204.82, 204.9,

	204.90, 204.91, 204.92, 205, 205.0, 205.00, 205.01, 205.02, 205.1, 205.10, 205.11, 205.12, 205.2, 205.20, 205.21, 205.22, 205.3, 205.30, 205.31, 205.32, 205.8, 205.80, 205.81, 205.82, 205.9, 205.90, 205.91, 205.92, 206, 206.0, 206.00, 206.01, 206.02, 206.1, 206.10, 206.11, 206.12, 206.2, 206.20, 206.21, 206.22, 206.8, 206.80, 206.81, 206.82, 206.9, 206.90, 206.91, 206.92, 207, 207.0, 207.00, 207.01, 207.02, 207.1, 207.10, 207.11, 207.12, 207.2, 207.20, 207.21, 207.22, 207.8, 207.80, 207.81, 207.82, 208, 208.0, 208.00, 208.01, 208.02, 208.1, 208.10, 208.11, 208.12, 208.2, 208.20, 208.21, 208.22, 208.8, 208.80, 208.81, 208.82, 208.9, 208.90, 208.91, 208.92, 209, 209.0, 209.00, 209.01, 209.02, 209.03, 209.1, 209.10, 209.11, 209.12, 209.13, 209.14, 209.15, 209.16, 209.17, 209.2, 209.20, 209.21, 209.22, 209.23, 209.24, 209.25, 209.26, 209.27, 209.29, 209.3, 209.30, 209.31, 209.32, 209.33, 209.34, 209.35, 209.36, 209.4, 209.40, 209.41, 209.42, 209.43, 209.5, 209.50, 209.51, 209.52, 209.53, 209.54, 209.55, 209.56, 209.57, 209.6, 209.60, 209.61, 209.62, 209.63, 209.64, 209.65, 209.66, 209.67, 209.69, 209.7, 209.70, 209.71, 209.72, 209.73, 209.74, 209.75 and 209.79<br><br>Other cancers<br>Diagnosis codes (index or prior admissions)<br>190, 190.0, 190.1, 190.2, 190.3, 190.4, 190.5, 190.6, 190.7, 190.8, 190.9, 191, 191.0, 191.1, 191.2, 191.3, 191.4, 191.5, 191.6, 191.7, 191.8, 191.9, 192, 192.0, 192.1, 192.2, 192.3, 192.8, 192.9, 193, 194, 194.0, 194.1, 194.3, 194.4, 194.5, 194.6, 194.8, 195, 195.0, 195.1, 195.2, 195.3, 195.4, 195.5, 195.8, 196, 196.0, 196.1, 196.2, 196.3, 196.5, 196.6, 196.8, 196.9, 197, 197.0, 197.1, 197.2, 197.3, 197.4, 197.5, 197.6, 197.7, 197.8, 198, 198.0, 198.1, 198.2, 198.3, 198.4, 198.5, 198.6, 198.7, 198.8, 198.81, 198.82, 198.89, 199, 199.0, 199.1, and 199.2
**Metastasic solid tumor**	Diagnosis Codes<br>190, 190.0, 190.1, 190.2, 190.3, 190.4, 190.5, 190.6, 190.7, 190.8, 190.9, 199, 199.0, 199.1, 199.2, 229.0, 229.8, 229.9, 238.9, 239.9
**Sepsis**	Diagnosis Codes<br>038, 038.0, 038.10, 038.11, 038.12, 038.19, 038.2, 028.3, 038.4, 038.40, 038.41, 038.42, 038.43, 038.44
**Shock**	Diagnosis Codes<br>785.5, 785.50, 785.51, 785.52, 785.59
**Acute Myocardial Infarction**	Diagnosis Codes<br>410, 410.0, 410.00, 410.01, 410.02, 410.1, 410.10, 410.11, 410.12, 410.2, 410.20, 410.21, 410.22, 410.3, 410.30, 410.31, 410.32, 410.4, 410.40, 410.41, 410.42, 410.5, 410.50, 410.51, 410.52, 410.6, 410.60, 410.61, 410.62, 410.7, 410.70, 410.71, 410.72, 410.8, 410.80, 410.81, 410.82, 410.9, 410.90, 410.91, 410.92, 412, 414.8
**Heart Failure**	Diagnosis Codes<br>428, 428.0, 428.1, 428.2, 428.20, 428.21, 428.22, 428.23, 428.3, 428.30, 428.31, 428.32, 428.33, 428.4, 428.40, 428.41, 428.42, 428.43, 428.9
**Stroke**	Diagnosis Codes<br>430, 431, 432, 432.0, 432.1, 432.9, 433, 433.0, 433.01, 433.1, 433.11, 433.2, 433.21, 433.3, 433.31, 433.8, 433.81, 433.9, 433.91,<br>434, 434.01, 434.1, 434.11, 434.9, 434.91, 435, 435.0, 435.1, 45.2, 435.3, 435.8, 435.9, 436

**Hemiplegia**	Diagnosis Codes<br>342, 342.0, 342.00, 342.01, 342.02, 342.1, 342.10, 342.11, 342.12, 342.8, 342.80, 342.81, 342.82, 342.9, 342.90, 342.91, 342.92
**Dementia**	Diagnosis Codes<br>294, 294.0, 294.1, 294.10, 294.11, 294.2, 294.20, 294.21, 294.8, 294.9
**Congenital Heart Disease**	Diagnosis Codes<br>745.10, 745.11, 745.12, 745.19, 745.2, 745.3, 745.4, 745.5, 745.6, 745.60, 745.61, 745.69, 745.7, 745.8, 745.9, 746.00, 746.01, 746.02, 746.09, 746.1, 746.2, 746.3, 746.4, 746.5, 746.6, 746.7, 746.8, 746.81, 746.82, 746.83, 746.84, 746.85, 746.86, 746.87, 746.89, 746.9, 747.0, 747.1, 747.10, 747.11, 747.2, 747.20, 747.21, 747.22, 747.29, 747.3, 747.31, 747.32, 747.39, 747.4, 747.40, 747.41, 747.42, 747.49, 747.5, 747.9, V13.65
**History of Drug Abuse**	Diagnosis Codes<br>304.0, 304.00, 304.01, 304.02, 304.03, 304.1, 304.10, 304.11, 304.12, 304.13, 304.2, 304.20, 304.21, 304.22, 304.23, 304.3, 304.30, 304.31, 304.32, 304.33, 304.4, 304.40, 304.41, 304.42, 304.43, 304.5, 304.50, 304.51, 304.52, 304.53, 304.6, 304.60, 304.61, 304.62, 304.63, 304.7, 304.70, 304.71, 304.72, 304.73, 304.8, 304.80, 304.81, 304.82, 304.83, 304.9, 304.90, 304.91, 304.92, 304.93
**Complications due to Cardiac Device**	Diagnosis Codes<br>996.0, 996.00, 996.01, 996.02, 996.03, 996.04, 996.09<br>996.1, 996.2, 996.3, 996.30, 996.31, 996.32, 996.39<br>996.4, 996.40, 996.41, 996.42, 996.43, 996.44, 996.45, 996.46, 996.47, 996.49, 996.5, 996.51, 996.52, 996.53, 996.54, 996.55, 996.56, 996.57, 996.59, 996.6, 996.60, 996.61, 996.62, 996.63, 996.64, 996.65, 996.66, 996.67, 996.68, 996.69, 996.7, 996.70, 996.71, 996.72, 996.73, 996.74, 996.75, 996.76, 996.77, 996.78, 996.79, 996.8, 996.80, 996.81, 996.82, 996.83, 996.84, 996.85, 996.86, 996.87, 996.88, 996.89, 996.9, 996.90, 996.91, 996.92, 996.93, 996.94, 996.95, 996.96, 996.99
**History of pacemaker or defibrillator placement**	Diagnosis Codes<br>V45.0, V45.00, V45.01, V45.02, V45.09, V53.3, V53.31, V53.32, V53.39, V45.0, V45.00, V45.01, V45.02, V4.09, V53.3, V53.31, V53.32, V53.39, 996.0, 996.00, 996.01, 996.02, 996.03, 996.04, 996.09
**History of prosthetic valve replacement**	Diagnosis Codes<br>V43.2, V43.21, V43.22, V43.3, 996.02, 996.71
**Hemodialysis**	Diagnosis Codes<br>403.0, 403.00, 403.01, 403.1, 403.10, 403.11, 403.9, 403.90, 403.91, 404.02, 404.03, 404.12, 404.13<br>404.92, 404.93, 585.6, V45.1, V45.11, V45.12, V56.0, V56.1, V56.3, V56.31
**Receiving intravenous therapy or home care**	Diagnosis Codes<br>V55, V55.0, V55.1, V55.2, V55.3, V55.4, V55.6, V55.7, V55.8, V55.9, V56.1, V58.11, V58.12, V58.62, V58.81, V66.2, V67.2, 996.62, 996.73, 996.74, 999.3, 999.31, 999.32, 999.33, 999.39
Causative Organisms	
Staphylococcus	Diagnosis Codes<br>0381, 03810, 03811, 03812, 03819, 0411, 04111, 04112, 0410 and 0419

Staphylococcus aureus	Diagnosis Codes<br>03811, 03812, 04111, and 0411
**Methicillin-resistant**	Diagnosis Codes<br>03812, 04112 (After 2008)<br>03811 or 04111 with either V090 or V091 (Before 2008)
**Streptococcus**	Diagnosis Codes<br>0380, 0382, 0410, 04100, 04101, 04102 , 04103, 04104, 04105, 04109 and 0412
**Gram-negative bacilli**	Diagnosis Codes<br>0384, 03840, 03841, 03842, 03843, 03844, 03849, 0413, 0414, 0415, 0416, 0417 and 04185
**Fungus**	Diagnosis Codes<br>1125, 11281, 1160, 11504, 11514, 11594 and 1173

Supplementary Table 2. Incidence of infective endocarditis classified by North, Center and South.

	Global	1997-1999	2000-2004	2005-2009	2010-2014	*p* value
**North**	2.47	1.96	2.51	2.54	2.66	< 0.001
**Center**	1.58	1.46	1.48	1.63	1.67	< 0.001
**South**	1.55	1.08	1.59	1.70	1.60	< 0.001

The units of measure for incidence are cases per 100,000 habitants.

Supplementary Table 3. Hazard Ratio of infective endocarditis stratified by age group and sex from 1997 to 2014.

	HR (95% CI)	*p*-value
Women	0.45 (0.44-0.46)	< 0.001
<5	0.19 (0.17-0.22)	< 0.001
5-9	0.07 (0.05-0.09)	< 0.001
10-14	0.09 (0.07-0.11)	< 0.001
15-19	0.14 (0.12-0.17)	< 0.001
20-24	0.24 (0.21-0.28)	< 0.001
25-29	0.41 (0.37-0.46)	< 0.001
30-34	0.57 (0.51-0.63)	< 0.001
35-39	0.58 (0.52-0.64)	< 0.001
40-44	0.63 (0.57-0.70)	< 0.001
45-49	0.75 (0.68-0.82)	< 0.001
50-54	Ref	
55-59	1.41 (1.30-1.52)	< 0.001
60-64	2.04 (1.89-2.20)	< 0.001
65-69	2.79 (2.60-2.99)	< 0.001
70-74	3.86 (3.61-4.13)	< 0.001
75-79	4.81 (4.50-5.15)	< 0.001
80-84	4.71 (4.40-5.03)	< 0.001
>=85	3.29 (3.07-3.53)	< 0.001

Supplementary figure 1. Hazard Ratio of infective endocarditis stratified by age group from 1997 to 2014.

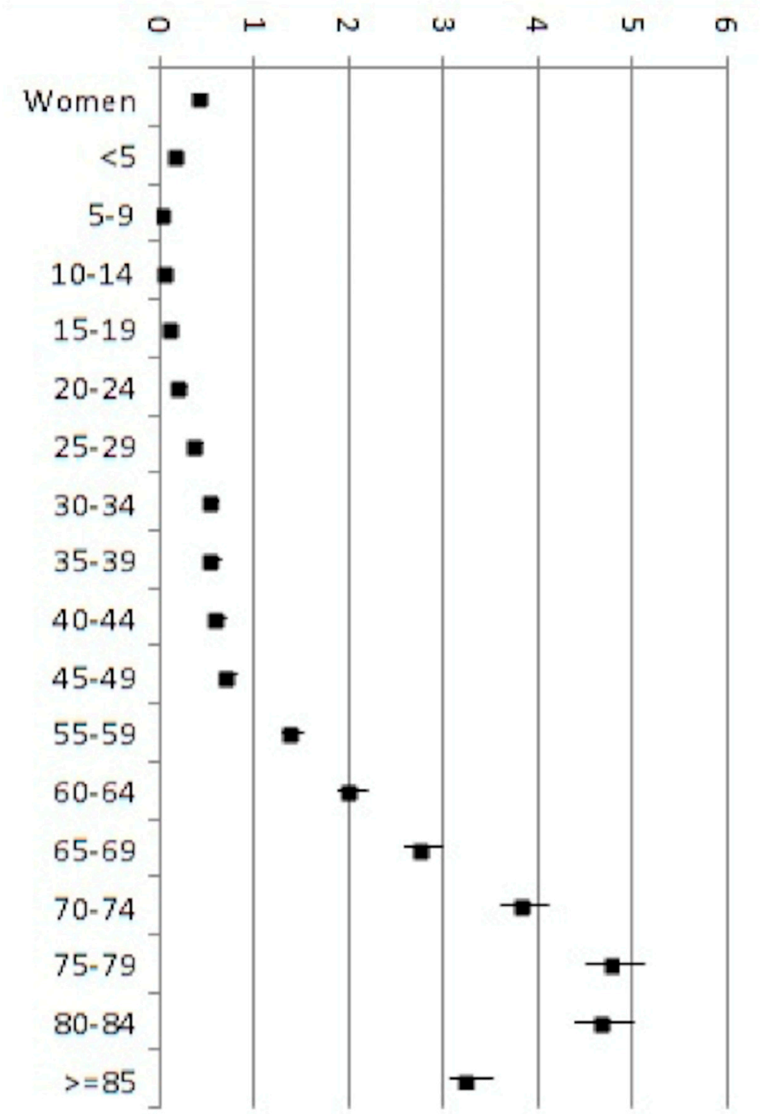

The units of measure for incidence are cases per 100,000 habitants per year. Error bars indicate 95% confidence intervals.
